# Primary Carcinoma of the Epiglottis, with Report of a Case

**Published:** 1913-09

**Authors:** Emil Mayer

**Affiliations:** New York


### Primary Carcinoma of the Epiglottis, With Report of a Case.

Primary cancer of the epiglottis is a rare disease, and up to the present time the treatment has consisted of surgical intervention or the use of radium and the X-ray. I have seen two cases of cancer of the epiglottis. The first case was diagnosed as probable malignant disease of the epiglottis, and the subsequent history was that of primary epithelioma of the larynx with subsequent metastatis, laryngectomy, and death. The second case was that a man, 64 years of age, who consulted me for some slight difficulty in swallowing, especially the swallowing of cold liquids. His general condition was good. Examination showed some edema of the uvula, and numerous small white spits on the tongue and inner surface of the cheek. The condition was diagnosed as leukoplakia buccalis. He gave no history of lues. After treatment he improved somewhat, but this did not last, and some six or seven months later there was discomfort in swallowing, a nasopharyngeal catarrh, and the white patches still present. There were no other evidences of a diseased condition in the throat. About three weeks later a new condition presented itself on the laryngeal surface of the epiglottis. There was a deep ulceration surrounded by a thickened mass. The pathologist pronounced this cylindrical celled carcinoma. Here I had a case of carcinoma of the epiglottis in its earliest stages. The epiglottis was removed, after the patient had been anesthetized and the suspension laryngoscope placed in position. There was little bleeding at the time of the operation, but twenty-six hours after, the patient began to expectorate large quantities of blood. This bleeding was checked by the application of ice internally and by sprays of peroxid of hydrogen. He left the hospital in ten days. This was the first operation of its kind ever performed under the ingenious suspension larygoscope of Killian. —Dr. Emil Mayer, New York.

